# Anomalous Right Coronary Artery: Culprit or Innocent Bystander?

**DOI:** 10.1155/cric/1450803

**Published:** 2025-01-04

**Authors:** Cooper B. Kersey, Shakirat Oyetunji, Creighton W. Don

**Affiliations:** ^1^Department of Medicine, Division of Cardiology, University of Washington, Seattle, Washington, USA; ^2^Department of Surgery, Division of Cardiothoracic Surgery, University of Washington, Seattle, Washington, USA

**Keywords:** anomalous coronary artery, aortic stenosis, heart team discussion

## Abstract

Anomalous aortic origin of a coronary artery is a rare congenital heart defect. The detection of anomalous coronary arteries is likely to increase with increased availability and application of cardiac computed tomography and magnetic resonance imaging. Once detected, the recommendation for surgical intervention on anomalous coronary arteries depends upon patient symptoms, the presence or absence of inducible ischemia on stress imaging, and high-risk anatomic features. A 77-year-old man with a history of hypertension, hyperlipidemia, chronic kidney disease Stage III, and moderate aortic stenosis presented with a non-ST-elevation myocardial infarction and was found to have an anomalous aortic origin of the right coronary artery on cross-sectional imaging. His aortic stenosis had also progressed from moderate to severe, and it was not clear whether his myocardial infarction could be exclusively attributed to a supply–demand disparity within the context of profound aortic stenosis or if his aberrant coronary anatomy could be implicated as the culprit for his presentation. A multidisciplinary heart team decided to proceed with a transcatheter aortic valve replacement and then readdress surgical intervention on his anomalous right coronary artery if his anginal symptoms persisted following valve replacement.

## 1. Introduction

Anomalous aortic origin of a coronary artery (AAOCA) is a rare condition with a prevalence of 0.03% for anomalous left coronary arteries off the right cusp and 0.23% for right coronary arteries off the left cusp [1, 2]. The detection of AAOCA is likely to increase with increased availability and application of cardiac computed tomography (CT) and magnetic resonance imaging (MRI). Once detected, the recommendation for surgical intervention depends upon patient symptoms, the presence or absence of inducible ischemia on stress imaging, and high-risk anatomic features [3]. We present the case of an older man with an anomalous aortic origin of the right coronary artery (AAORCA) who presented with a non-ST-elevation myocardial infarction (NSTEMI) to illustrate the nuances of the assessment of anomalous coronary arteries.

## 2. Case Presentation

A 77-year-old man with a history of hypertension, hyperlipidemia, chronic kidney disease Stage III, and moderate aortic stenosis (aortic valve area (AVA) 0.9 cm^2^, dimensionless valve index (DVI) 0.28, peak velocity 3.6 m/s, mean gradient 37.8 mmHg) presented with acute onset substernal chest pain that occurred while moving furniture in his house and persisted at rest. On exam, he was hypertensive to 174/89 mmHg, in sinus rhythm at 60 beats per minute, and with an oxygen saturation of 100% on ambient air. Cardiac auscultation revealed a 3/6 systolic ejection murmur best heard at the right upper sternal border. His initial troponin was less than 0.03 ng/mL and then trended upwards to a peak of 3.75 ng/mL 18 h later. His electrocardiogram revealed diffuse 1-mm ST depressions, and he was admitted for management of his NSTEMI.

**Figure 1:**
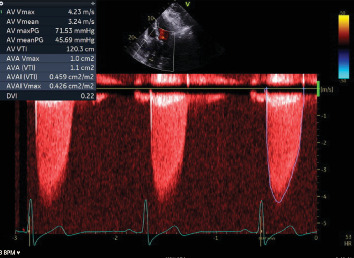
Continuous wave Doppler tracing across aortic valve demonstrating severe aortic stenosis.

**Figure 2:**
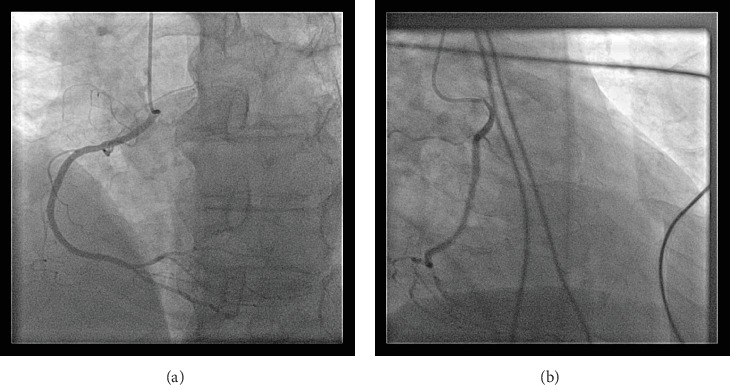
(a) Left anterior oblique coronary angiogram of the right coronary artery demonstrating the high anterior origin of the coronary. (b) Right anterior oblique coronary angiogram of the right coronary artery demonstrating the high anterior origin of the coronary.

**Figure 3:**
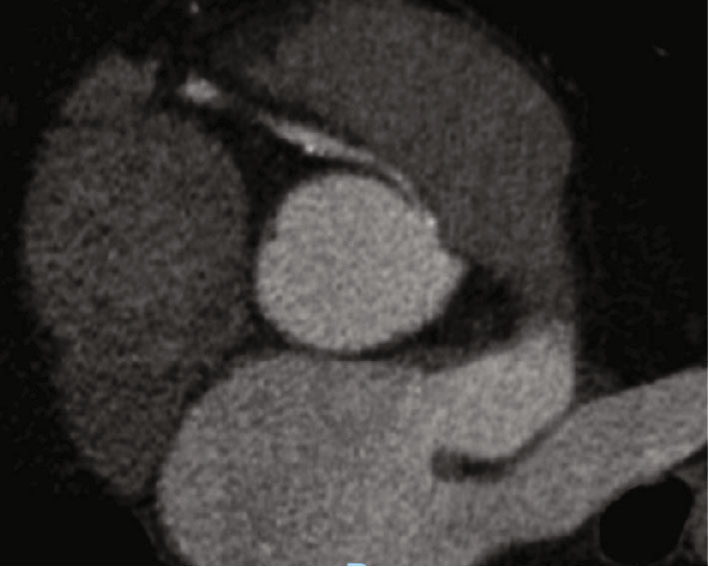
CT angiogram reveals the anomalous origin of the right coronary artery of the left coronary cusp with a slit-like opening and an interarterial course.

A transthoracic echocardiogram demonstrated that his aortic stenosis had progressed from moderate to severe (AVA 0.8 cm^2^, DVI 0.22, peak velocity 4.2 m/s, mean gradient 45.7 mmHg) (Figure 1). The patient underwent a coronary angiogram which showed no obstructive coronary artery disease. During the procedure, his right coronary artery (RCA) was challenging to engage with both the Judkins Right 4 (JR4) and Jacky catheters. The RCA was eventually engaged with an Amplatz Right 2 (AR2) catheter and was noted to have a high and anterior origin (Figures 2(a) and 2(b)) For the planning of his aortic valve replacement, he underwent a CT transcatheter aortic valve replacement (TAVR) protocol which revealed an anomalous origin of the RCA off of the left coronary cusp (Figure 3). The RCA had a slit-like orifice and took an interarterial course, but there was no clear evidence of vessel compression. The coronary height (leaflet distance to coronary ostia) for both the RCA and left main coronary artery was 14 mm.

The patient was discussed at our institution's multidisciplinary heart team conference for consideration of transcatheter versus surgical aortic valve replacement. One aspect of his clinical presentation that remained ambiguous was the determination of whether his NSTEMI could be exclusively attributed to a supply–demand disparity within the context of profound aortic stenosis or if his aberrant coronary anatomy could be implicated as the driver of his myocardial infarction. Coronary artery compression was an important consideration for procedural planning because anomalous coronary arteries are best addressed surgically with either unroofing of the intramural segment of the anomalous artery, reimplantation of the native RCA onto the right sinus of the aortic root, or coronary artery bypass grafting. While the vessel was patent on CT imaging, the interarterial course and slit-like opening made it a high risk for compression. A potential mechanism of dynamic compression that was posited was that he could have exertional pulmonary hypertension leading to pulmonary artery dilation and compressive symptoms. Ultimately, the patient's functional capacity was limited by his severe aortic stenosis to the point that provocative exercise testing to assess for coronary artery compression could not be performed. The decision was made to perform TAVR for his aortic stenosis and then assess whether he had anginal symptoms despite addressing his severe valvular disease.

**Figure 4:**
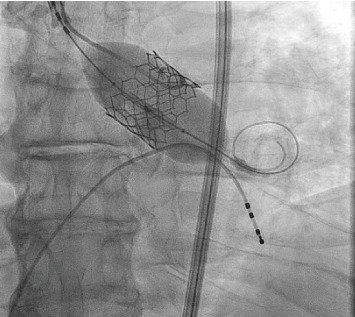
Balloon expansion of the 29-mm Sapien 3 valve.

The patient underwent a successful TAVR (Figure 4) and reports feeling a significant improvement in symptoms following valve intervention. He is free of chest pain, and his exertional tolerance has improved. At 1 year follow-up, the patient continues to note that he is chest pain-free and ambulatory.

## 3. Discussion

Once an AAOCA is identified, the subsequent work-up is focused on stratifying the lesion's risk of causing sudden cardiac death based on its anatomic characteristics and functional significance. Anomalous aortic origins of the left coronary artery (AAOLCA) have a higher incidence of sudden cardiac death due to supplying a larger region of the myocardium. Anatomic features that increase the risk of coronary artery compression are, if the vessel takes an interarterial course, the length of the intramural course of the vessel, the presence of a slit-like opening, and an acute angle take-off of the vessel. Our patient had two of these high-risk features (interaterial course and a slit-like opening) on cross-sectional imaging which raised our clinical suspicion for compression.

The European Society of Cardiology (ESC) guidelines for the management of adult congenital heart disease as well as other society guidelines recommend testing for exercise-induced ischemia with advanced imaging modalities in patients with AAOCA [3–5]. For AAORCA, surgery is recommended if patients have either angina or stress-induced myocardial ischemia on physiologic testing. Due to the higher risk of sudden death with AAOLCA, surgery is also recommended for asymptomatic patients who are young (less than 35 years old) or asymptomatic patients with high-risk anatomic features. The presence of both symptoms and inducible ischemia garners a Class I recommendation, whereas intervention based on symptoms, physiologic testing, or anatomic features alone receives a IIa or IIb recommendation. Exercise stress testing without imaging is useful in ruling in patients who would benefit from surgical intervention, but a large cohort study of 220 pediatric patients demonstrated that exercise stress testing alone (without imaging) is a poor predictor of which patients are at low risk of sudden cardiac death [6]. The clinical algorithm at Texas Children's Hospital mirrors the ESC recommendations and suggests combining exercise testing with advanced imaging like positron emission tomography (PET) or cardiac MRI to improve sensitivity.

Applying the ESC guidelines to our patient, he had ischemic symptoms on presentation, but this was confounded by his severe aortic stenosis. We were unable to further investigate whether he had inducible ischemia in the RCA distribution with an exercise stress test given his poor exercise tolerance. Furthermore, the multidisciplinary heart team considered the low likelihood that the patient would develop compressive symptoms for the first time in his eighth decade of life. A retrospective review of patients undergoing surgery for AAOCA due to ischemic symptoms or positive stress tests demonstrated a median age of 47 years old, with an age range from 13 to 82 years old [7]. A possible mechanism for the wide age range for patients developing ischemia is that both aortic and pulmonary artery size change with age and can cause anomalous coronaries to become more susceptible to compression over time. It is important to note that coronary artery occlusion during TAVR for a patient with AAOCA can be catastrophic in the sense that the deployment of the TAVR valve can obstruct both coronary ostia simultaneously. Our patient's coronary height (14 mm) on preprocedure CT scan was in the range where the coronary obstruction was less likely to occur.

We describe the case of an older patient with severe aortic stenosis who presented with an NSTEMI and was found to have an AAORCA on cardiac CT after undergoing a coronary angiogram devoid of a culprit lesion. This case highlights the importance of having a multidisciplinary heart team approach to complex patients with both coronary and valvular disease to discern whether a surgical or transcatheter approach is most clinically appropriate. With increasing volumes of cardiac CT and MRI being performed, cardiologists should be comfortable with the functional and anatomic work-up of anomalous coronary arteries and indications for surgical referral.

## Data Availability

All data referenced in the case report can be made available with reasonable request.

## Nomenclature

AAOCA: anomalous aortic origin of coronary artery
CT: computed tomography
MRI: magnetic resonance imaging
AAORCA: anomalous aortic origin of the right coronary artery
NSTEMI: non-ST-elevation myocardial infarction
TAVR: transcatheter aortic valve replacement
AVA: aortic valve area
DVI: dimensionless valve index
RCA: right coronary artery
JR4: Judkins Right 4 (catheter)
AR4: Amplatz Right 4 (catheter)
AAOLCA: anomalous aortic origin of the left coronary artery
ESC: European Society of Cardiology
PET: positron emission tomography
